# First person – Marek Hampl

**DOI:** 10.1242/dmm.050811

**Published:** 2024-06-20

## Abstract

First Person is a series of interviews with the first authors of a selection of papers published in Disease Models & Mechanisms, helping researchers promote themselves alongside their papers. Marek Hampl is first author on ‘ Early embryogenesis in CHDFIDD mouse model reveals facial clefts and altered cranial neurogenesis’, published in DMM. Marek is a postdoc in the lab of Marcela Buchtová at the Institute of Animal Physiology and Genetics, Brno, Czech Republic, investigating developmental defects of craniofacial region and limbs.


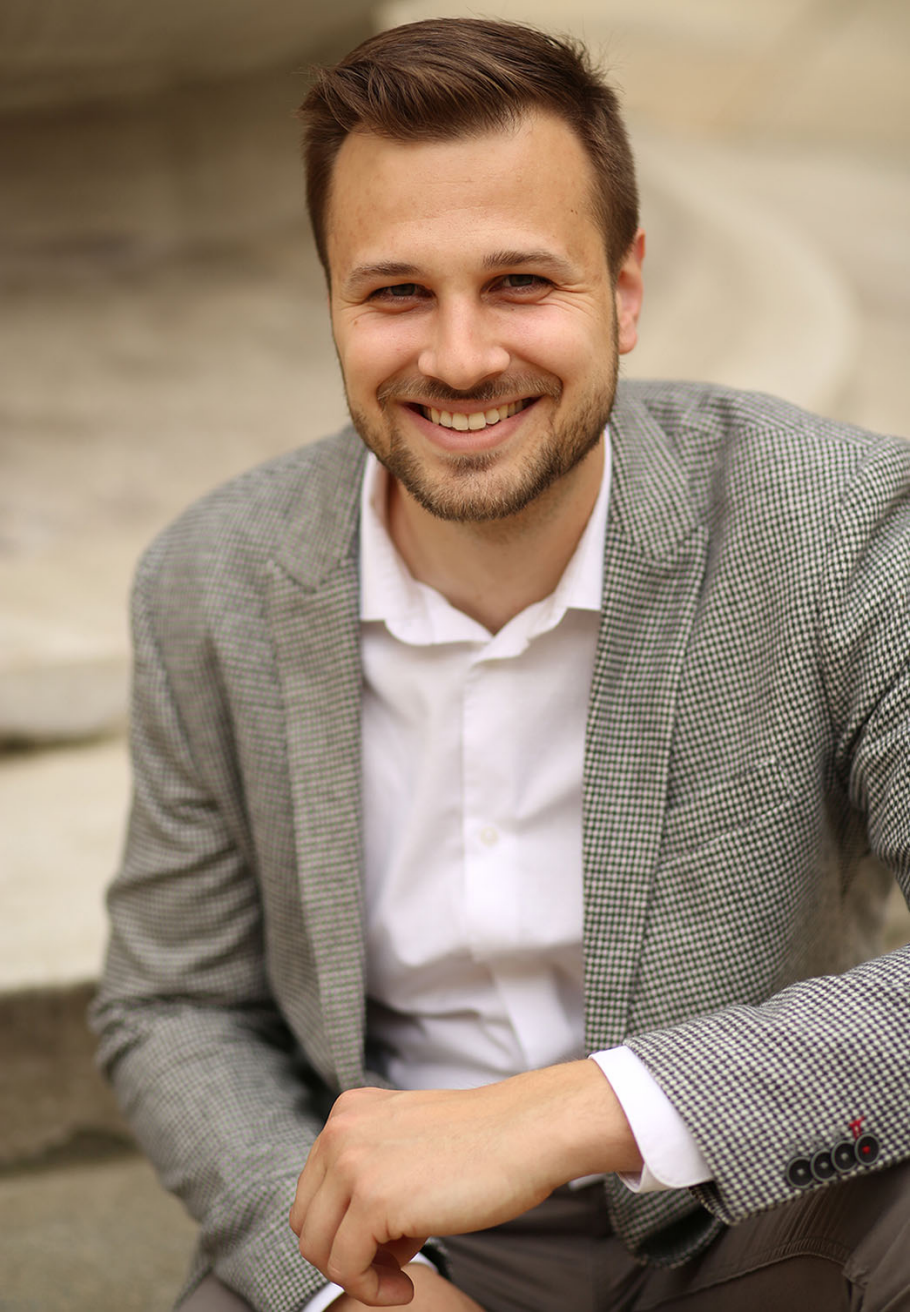


**Marek Hampl**

**How would you explain the main findings of your paper to non-scientific family and friends?**

Congenital heart defects, dysmorphic facial features and intellectual developmental disorder (CHDFIDD) caused by mutations in the *CDK13* gene is a rare disease first identified in 2016. Every year, a few more cases are detected, currently amounting to ∼300 cases worldwide. This makes CDK13 an important protein regulating gene transcription, a kinase with pleiotropic effects on the development of various tissues and organs. To study the importance of CDK13 in embryonic development during which altered CDK13 function often results in developmental defects, we use Cdk13-deficient mouse models with phenotypic manifestations comparable to those in human patients. Thanks to these mouse models, we have been able to analyse developmental craniofacial defects, and detect genes that are important for the formation of individual facial parts and cranial nerves, and are deregulated due to Cdk13 insufficiency.

Cdk13 is an important protein in the regulation of tissue-specific gene expression during embryonic development

**What are the potential implications of these results for your field of research?**

As CHDFIDD is a rare disease, research is a bit behind the general research investigating developmental defects. Our study shows that Cdk13 is an important protein in the regulation of tissue-specific gene expression during embryonic development, specifically observed in the morphogenesis of facial prominences and peripheral nerves that develop during formation of the craniofacial region. Our findings revealed that Cdk13 can regulate the expression of genes that are indispensable for development of the above mentioned structures, e.g. *Shh*, *Fgf8*, *Pou4f1* and *Neurog1*. Thus, Cdk13 is likely to be responsible for a more-general regulation at transcription level in a tissue-specific manner, and our mouse models can be used to further analyse the processes involved in the development of other defective tissues, e.g. brain, heart, liver or limbs.

**What are the main advantages and drawbacks of the experimental system you have used as it relates to the disease you are investigating?**

As an experimental system, we use hypomorphic as well as knockout *Cdk13* mouse models. Both show phenotypic manifestations very similar to those found in patients diagnosed with CHDFIDD and who carry *CDK13* mutations – including malfunction of the brain, heart, kidney, face, limbs, etc. – and, thus, are phenotypically valuable models. Moreover, the overall percentage of mutant embryos with a distinct phenotype is high. The main differences compared to human patients is their embryonic lethality – hypomorph around embryonic day 16 (E16) and knockout around E14 – and their different genotype, i.e. mouse mutants are homozygous, while human patients are heterozygous. Embryonic lethality, especially, would limit the use of mouse models if we were, for example, to conduct behavioural studies (intellectual developmental defects observed in humans diagnosed with CHDFIDD) in postnatal, juvenile or adult individuals. Luckily, such experiments are not in our area of expertise.

**Figure:**
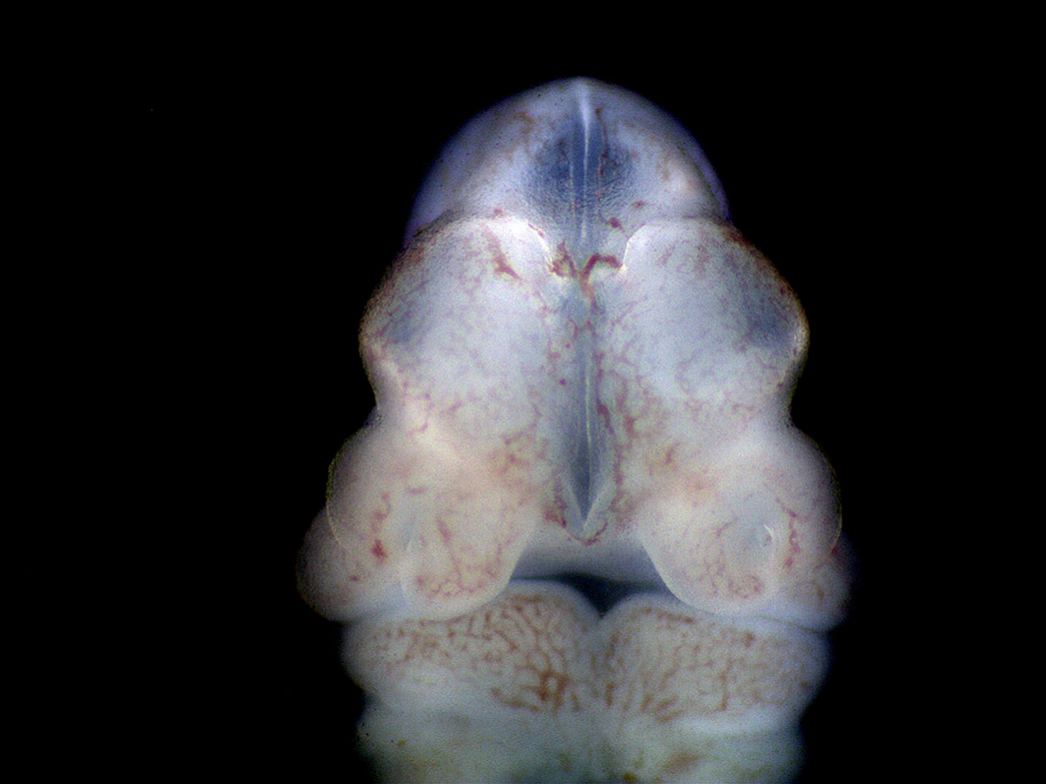
**Representative image showing the *Cdk13^tm1d/tm1d^* external craniofacial phenotype of a mouse embryo at E11.5.** Noticeable are the especially wide-set nasal prominences resulting in the midfacial cleft with a remarkably exposed developing telencephalon.

**What has surprised you the most while conducting your research?**

At the very beginning, we had planned to do some basic macroscopic and microscopic phenotyping of the craniofacial region in Cdk13-deficient mouse embryos, and perform gene and protein expression assays to find differences in the expression patterns of only those proteins most-important for craniofacial development. But, during our research (which lasted for almost 8 years, as the study was conducted as one of our side projects), we finally run into problems, i.e., we needed help from fellow researchers, who are experienced in additional molecular genetics methods and also in techniques used to track live cells in migration assays. This led us, finally, to learning and using techniques that are now part of our routine methodological portfolio.

**Describe what you think is the most significant challenge impacting your research at this time and how will this be addressed over the next 10 years?**

Our research focussing on the role of Cdk13 within the craniofacial region revealed, in our mouse models, the development of hypoplastic cranial nerves that poorly penetrate into individual facial prominences. In these mice, development of facial prominences is disturbed and results in cleft palate, cleft lip or midfacial cleft. The question is whether defects in the formation of the facial region emerge in response to insufficient growth of facial nerves or whether developmental defects of both facial region and cranial nerves are two independent defective processes happening together. Elucidating such non-canonical function of peripheral nerves during the development of the facial region could be, in my view, one of the significant challenges, which could impact research of craniofacial defects. A functional connection between defects of cranial nerve development – that, in turn, leads to facial malformations – has been detected only in few cases, i.e. for Möbius syndrome, hereditary sensory and autonomic neuropathy type IV (HSAN-IV) and Parry-Romberg syndrome (also known as progressive hemifacial atrophy).

**What changes do you think could improve the professional lives of scientists?**

On the one hand, based on the general scientific discussion, the major problem is, and probably will be over the next few years, the financial support of all research-related issues (personnel costs, expenses covering research material and, also, higher-education expenses). On the other hand, working conditions are much better than before, as we can work from home (home office), and use flexible working hours to adapt our working schedule to better combine family needs and work. From the perspective of a scientist at the start of the scientific career (I got my PhD in 2020 at the Masaryk University in Brno, Czech Republic), there are changes happening quickly especially in PhD programs. Students have lots of opportunities to attend many workshops and lectures, and also can use many new facilities offering state-of-the-art appliances and techniques, which can help them conduct their research projects.

**What's next for you?**

Currently, I am following the study of migration properties of cells in response to chemotactic stimuli from diverse environments. The other study I am involved in, is focused on anterior-posterior patterning defects of the developing limb buds by using embryonic mouse and chicken models.
